# Delayed Diagnosis of McCune–Albright Syndrome

**DOI:** 10.1155/2021/2999349

**Published:** 2021-12-10

**Authors:** Bereket Fantahun, Seblewongel Desta

**Affiliations:** Department of Pediatrics and Child Health, Saint Paul's Hospital Millennium Medical College, Addis Ababa, Ethiopia

## Abstract

**Background:**

McCune–Albright syndrome (MAS) is a rare heterogeneous genetic disorder that is characterized by a triad of polyostotic fibrous dysplasia (FD), café au lait spots (CAL), and multiple hyperfunctional endocrinopathies. In general, it is diagnosed clinically. From the triads, two of the findings are enough to make the diagnosis, but genetic testing can be done if it is available. *Case Presentation*. We report a female child who was symptomatic since the neonatal period with skin hyperpigmentation, breast enlargement, and vaginal bleeding. She was diagnosed with MAS at the age of five years. She had pathological fractures at multiple sites and had raised thyroid hormones since the age of 3½ years. The child developed severe morbidity as the result of delayed diagnosis and currently became wheelchair dependent.

**Conclusion:**

Thorough patient evaluation and appropriate interpretation of findings are crucial steps for timely diagnosis of MAS and better patient care outcomes.

## 1. Background

McCune–Albright syndrome (MAS) is a rare sporadic disease characterized by Fibrous Dysplasia (FD), café au lait (CAL) spots, and hyperfunctional endocrinopathies. The prevalence of MAS is estimated between 1/100,000 and 1/1,000,000 [1, 2]. Its manifestations are due to somatic activating mutations in the guanine nucleotide binding protein, alpha stimulating (GNAS) gene. The gene codes for alpha subunit of a stimulatory G-protein (Gs*α*) which is involved in intracellular cyclic adenosine monophosphate (cAMP) production. The mutation on the alpha subunit results in impaired guanosine triphosphatase activity, which causes persistent adenylate cyclase activation and cellular hyperfunction [2, 3].

There have been no reports of heritable MAS, as the mutation is lethal to the zygote. It is postulated that the disease is caused by an autosomal dominant lethal gene, leading to loss of the zygote in utero. Cells carrying the mutation can only survive when they are combined with normal cells [4]. The broad tissue distribution and the mosaic distribution of the Gs*α* mutation results in wide spectrum of extraskeletal manifestations with many organ involvements. Because of the wide distribution of the mutation, systematic screening of tissues is important [2, 5].

MAS is generally diagnosed based on clinical findings. Though the importance of genetic testing in a clear clinical diagnosis is uncertain, it can be done if it is available. Histological examination is only required in unusual or questionable cases. If there are uncertain histologic findings, confirmation by molecular tests is recommended [2, 6]. As MAS is very rare, we are reporting the condition which has never been reported from Ethiopia.

## 2. Case Presentation

We present a 5-year-old female child who was symptomatic since the early neonatal period with skin lesions, intermittent painless vaginal bleeding, and breast enlargement. For these complaints, the parents took the child to the nearby health facility, but they were reassured. Compared to her peers, her growth in length was fast since her early childhood, but she had poor weight gain. At the age of 3 ½ years, she presented to an orthopedic clinic with bowlegs for which she was seen and sent home without any intervention. But after one week, she had a trivial fall down accident, and she sustained pathological fractures on both upper and lower extremities. Plaster of Paris (POP) cast was applied for the lower left arm, and open fixation with plate was done for the left femur.

During the procedure, tachycardia was detected, for which she was investigated and diagnosed to have hyperthyroidism. She was initially put on propylthiouracil (PTU) and propranolol. After eight months of the procedure, there was displacement of the plate. The orthopedic surgeon decided to revise the operation, but the thyroid function was not controlled for which she was referred to a paediatric endocrinology clinic for better management of hyperthyroidism.

**Figure 1:**
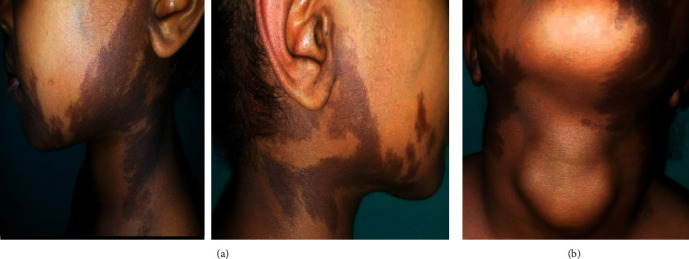
(a) Lesions on both sides of the face seen on a 5-year-old girl with McCune–Albright syndrome, which demonstrates irregular borders (coast of Maine). (b) Nodular goiter and the tendency for the café au lait lesions to respect the midline.

On physical examination at the paediatric endocrinology clinic, she was emaciated. Her weight was 16 kg (between 10^th^ and 25^th^ percentiles) and her height was 115 cm (on the 95^th^ percentiles). Weight for height was far less than 5^th^ percentile (underweight), based on CDC growth charts. Her pulse rate was 123 bpm, and she had protruded eyes. CAL spots were noticed on her face, neck, and trunk (Figure 1(a)). There was a 5 cm by 3 cm anterior neck mass with an irregular surface (Figure 1(b)). There was also breast enlargement. She had a grade III early systolic murmur best heard at the left upper sternal border. There was swelling and tenderness at the right midshaft of the humerus and short POP on the left lower arm. She had a wide gait. Based on her clinical findings, she was diagnosed to have MAS.

## 3. Investigation Results

### 3.1. Hormonal Analysis

Initial thyroid function test (TFT) results showed high T3 (3.36 ng/ml, normal 0.95–2.5 ng/ml) and T4 (14 mcg/dl, normal 6–13 mcg/dL) and low TSH (<0.015 *μ*lu/mL, normal 0.4–6.6 *μ*lu/mL) values, which were suggestive of hyperthyroidism. Serum cortisol level was normal (7.2 *μ*g/dL, normal 3.7–19.4 *μ*g/dL). Follicular stimulating hormone (FSH) 0.63 mIu/mL, luteinizing hormone (LH), and estradiol levels were undetectable, and all (FSH, LH, and estradiol levels) were in the prepubertal range.

Both calcium and phosphorus were normal previously, but the recent laboratory test showed slightly low level of Ca^++^ (2.12 mmol/L, normal 2.2–2.7 mmol/L) and phosphorus (1.17 mmol/L, normal 1.45–2.1 mmol/L) for her age. PTH (114.8 pg/mL, normal 10–55 pg/mL) and alkaline phosphatase (777 IU/L, normal 48.8–445.9 U/L) were both high for her age. These findings were suggestive of vitamin D deficiency with secondary hyperparathyroidism.

The urine phosphate excretion test was not done, since it is not available in the hospital.

### 3.2. Radiology Findings

**Figure 2:**
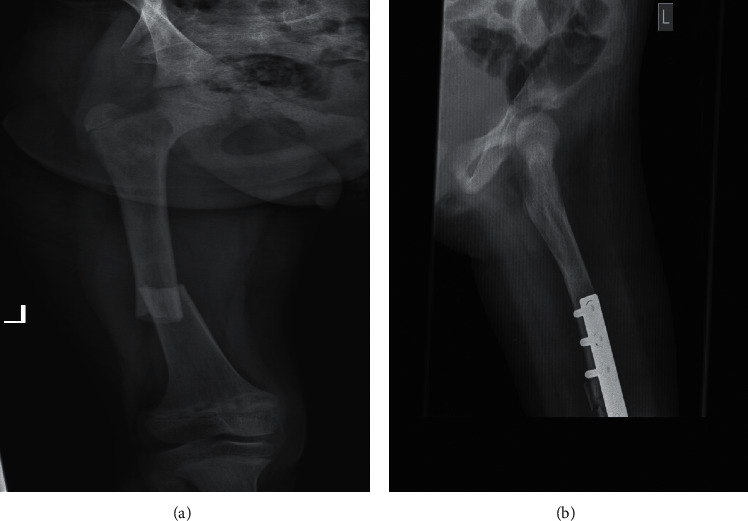
Radiologic appearance of fibrous dysplasia. (a) Left femur X-ray: there was intramedullary expansive lesion resulted in endosteal scalloping with ground glass and lucent matrix involving the femoral neck up to the midlower shaft of the femur. Pathological fracture is seen at the midlower shaft of the femur with overlapping fracture segments. (b) Posttreatment X-ray: callus formation seen at the site of the pathologic fracture.

Bone X-ray was suggestive of fibrous dysplasia (Figure 2). The radiologic report of the upper extremities also showed lesions of FD. Her bone age was 12 years. Thyroid ultrasound showed enlarged gland left lobe 4.5 × 1.5 × 1.8 cm and right lobe 3.6 × 1.2 cm with multiple small hypoechogenic nodules. Pelvic ultrasound showed bilateral ovarian cysts. Echocardiography showed atrial septal defect (ASD), mitral valve prolapse, and moderate mitral regurgitation.

### 3.3. Molecular and Genetic Tests

These tests were not done.

### 3.4. Treatment

Vitamin D and calcium supplementation was given. The child was taking carbimazole, and thyroid function tests were normalized. She was taken to Israel, and ASD was surgically repaired. While she was in Israel, she sustained right femoral fracture, and an intramedullary nail was inserted.

## 4. Discussion

MAS is a rare sporadic genetic disorder which can affect many tissues [2]. The success of treatment of this disease directly depends on early diagnosis and timely referral of the patient to an appropriate institution. But the rarity of the disease and its variable presentations lead to delayed diagnosis, inappropriate investigations, and delayed treatment with many complications [6, 7]. Because of the rarity of the disease, only few individuals are reported [8]. We are reporting a child who was diagnosed with MAS at the age of five years with multiple organ involvement since her early childhood. As the disease affects many organs, its management requires multidisciplinary approach [6].

FD is one of the major manifestations of the syndrome which results in bone pain, bone deformity, and bone fracture, which can lead into functional impairment and wheel chair confinement [9]. The disease process might be localized to one bone or can affect multiple bones. It is usually diagnosed by radiologic findings [10]. There is no cure for FD, but there are options to manage FD. Exercise and rehabilitations are recommended to optimize the strength and function of the bones. Patients can also be supplemented with calcium and vitamin D [9]. When there is fracture, it is recommended to insert intramedullary nail or a special angled base plate to stabilize the bone and to prevent uncontrolled fracture [6]. The case reported here presented with bowlegs and multiple site fractures. She was managed by applying POP cast and internal fixation which were not the recommended procedures in FD in patients with MAS. Despite the procedures and the supportive management, the child had severe bone deformities and confined to wheelchair. These were due to the delayed diagnosis coupled with the late and inappropriate interventions.

Another manifestation of the syndrome is CAL spots, which occur as the result of increased melanin production in mutation-bearing cells. They are hyperpigmented macular lesions that may vary in color from light brown to dark brown and with characteristic features of jagged, irregular borders (coast of Maine) and a distribution showing the so-called “respect of” the midline of the body as it was observed in our patient. They can be an early clue to the diagnosis of MAS and are typically the first manifestation of the syndrome, usually appearing either at or shortly after birth as it was demonstrated in the current report [5, 6].

The involvement of the endocrine system is another part of the triad of MAS, of which gonadotropin-independent precocious puberty (PP) is the predominating and common initial presentation of MAS among affected children [11, 12]. Girls are more affected than boys [7]. PP in girls is caused by the sporadic development of ovarian cysts producing estrogens [11]. As it was evidenced in this report, PP typically presents in girls in early childhood with painless vaginal bleeding and breast enlargement [7, 11]. The peripheral PP seen in MAS can progress into central PP. Because of this, the treatment of PP in MAS is important in order to decrease estrogen exposure with the objective of preventing vaginal bleeding, decreasing psychological distress, halting pubertal progression, and improving adult height [12–15]. Ovarian surgery for cysts should be avoided unless there is a risk of torsion. If there is advanced bone age, frequent vaginal bleeding treatment is indicated. First line suggested drug is letrozole with tamoxifen, and patients should be monitored for central puberty and the need to add a gonadotropin releasing hormone analogue (GnRHa), like leuprolide [6]. Both patients as well as their families need continuous psychological support. The child in this report was not yet started on treatment as the bleeding was infrequent, but recently, she had advanced bone age and planned to start GnRHa.

Hyperthyroidism is regarded as the second most common endocrine manifestation in MAS. The mutation in the thyroid gland results in hyperplasia and hyperfunction of the thyroid gland, and it also increases the deiodinase activity which causes T3-dominant hyperthyroidism [1, 6]. Early diagnosis and treatment of hyperthyroidism is important. The hyperthyroidism responds well for the antithyroidal drugs, but spontaneous resolution is unlikely to occur. Therefore, surgical or medical ablation might be required [16, 17]. Hyperthyroidism was the second endocrine abnormality which was seen in the current report. Initially, she was taking PTU which was later changed to carbimazole, and the patient was advised to have surgery.

There are also other extraskeletal manifestations of MAS such as renal phosphate wasting syndrome, Cushing syndrome, and acromegaly. Renal phosphate wasting syndrome occurs as a result FGF-23 mediated hypophosphatemia. In the current study, though the child had hypophosphatemia, it was not possible to determine the urine excretion of phosphorus and creatinine and to make a conclusion. The adrenal involvement in patients with MAS results in hypercortisolism, which occurs exclusively in the first year of life. But in the current patient, the serum cortisol level was normal, and there was no history of adrenal involvement during infancy. Though IGF-1 was not determined, there were no clinical findings suggestive of acromegaly. In summary, as patients with MAS have multiorgan involvement, they should be screened systematically for potential tissues involvement [5, 6].

Because of the rarity of the condition, the child was diagnosed late. This puts her in severe morbidity as it was also seen in another case report from Africa [18].

## 5. Conclusion

MAS in our patient involved multiple organs mirroring previous reports. As the syndrome has wide spectrum manifestations, systematic screening is crucial. The condition requires multidisciplinary approach and regular follow-up in order to minimize severe complications. This case report urges health professionals to move from organ specific to overall patient approach in order to make appropriate disease diagnosis and timely patient care.

## Data Availability

The data used to support this study are included within the article.

## Abbreviations

ASD: Atrial septal defect
CAL: Café au lait
GNAS: Guanine nucleotide-binding protein alpha stimulating
Gs*α*: Alpha subunit of a stimulatory G-protein
cAMP: Cyclic adenosine monophosphate
FD: Fibrous dysplasia
FSH: Follicular stimulating hormone
LH: Luteinizing hormone
MAS: McCune-Albright syndrome
POP: Plaster of Paris
PP: Precocious puberty
PTU: Propylthiouracil
SPHMMC: Saint Paul's Hospital Millennium Medical College
TFT: Thyroid function test
TSH: Thyroid stimulating hormone.
